# Genetic Diversity Assessment and Marker-Assisted Selection in Crops

**DOI:** 10.3390/genes11121481

**Published:** 2020-12-09

**Authors:** Francesco Mercati, Francesco Sunseri

**Affiliations:** 1 CNR—Consiglio Nazionale delle Ricerche, Istituto di Bioscienze e Biorisorse (IBBR), Corso Calatafimi 414, 90129 Palermo, Italy; 2 Dipartimento di Agraria, Università Mediterranea degli Studi di Reggio Calabria, Loc. Feo di Vito, 89124 Reggio Calabria, Italy

Global warming is negatively impacting on crop yield and Earth’s climate changes can bring possible negative effects on the growth and reproductive success of crops. Therefore, the exploitation of biodiversity is essential to select more resilient genotypes employable in more sustainable cropping systems.

The assessment of genetic diversity from the major crops and their wild relatives together with its exploitation have been always among the main challenges for plant breeding, as recently highlighted [1,2]. The wide utilization of molecular markers for mapping traits of agronomic interest in specific genomic regions appears to return back another pivotal effort for the future development of novel cultivars [3]. Indeed, the improvement of plant breeding efficacy has always gone through the construction of exotic genetic libraries, exploiting the genetic resources [4].

Nowadays, there is evidence that MAGIC and other exotic populations will play a major role in the coming years in allowing for impressive gains in plant breeding for developing new generations of improved cultivars [5].

This Special Issue focused on the application of such advanced technologies devoted to crop improvement, exploiting the available biodiversity in crops. In detail, next-generation sequencing (NGS) technologies supported the development of high-density genotyping arrays for different plants included in this issue.

By using a high throughput approach, here we report a new high-resolution eggplant (*Solanum melongena* L.) genetic map based on a RIL population and Genotyping by Sequencing (GBS) analysis by which 7249 SNPs were assigned to the 12 chromosomes spanning 2169.23 cM [6]. Afterwards, the phenotyping of the RIL population at three locations allowed us to elucidate the genetic bases of seven traits related to anthocyanin content in eggplant as well as seed vigor. Overall, between 7 and 17 QTLs (at least one major QTL) were identified for each trait [6]. Otherwise, a genome-wide association scan (GWAS) using 121 accessions and a 9K single nucleotide polymorphisms (SNPs) chip were also reported to clarify the genetic determinants underlying drought tolerance in barley (*Hordeum vulgare* L.) [7]. Overall, a total number of 101 significant SNPs, distributed over all seven barley chromosomes, were found to be highly associated with the studied traits, of which five genomic regions were associated with candidate genes at chromosomes 2 and 3 [7].

The limited availability of simple sequence repeats (SSR) in *Paeonia lactiflora*, a flowering crop with great economic value, triggered a study to develop a novel SSR panel with Illumina RNA sequencing for also assessing the role of these variants in gene regulation. The results showed that dinucleotides with AG/CT repeats were the most abundant type of repeat motif in *P. lactiflora* and were preferentially distributed in untranslated regions. Significant differences in SSR size were observed among motif types and locations [8]. This new set of SSRs will aid programs for accession identification, marker-trait association and molecular assisted breeding in *P. lactiflora* [8].

QTL-related Lethal Necrosis (LN) tolerance/resistance in maize (*Zea mays* L.) has been studied by using five hundred selected kompetitive allele specific PCR (KASP) SNPs and multiple mapping populations [9]. To understand the status of previously identified quantitative trait loci (QTL) in diverse genetic backgrounds, F_3_ progenies derived from seven bi-parental populations were genotyped and phenotyped under artificial LN inoculation for three seasons. Joint linkage association mapping revealed at least seven major QTL spread across the 7-biparetal populations, for resistance to LN infections potentially useful for marker-assisted breeding [9].

A particular resequencing approach was utilized for exploring the natural variation and the domestication selection of *ZmPGP1*, involved in the polar auxin transport and associated to plant height, leaf angle, yield traits, and root development in maize (*Z. mays* L.) [10]. Li et al. (2019) [10] re-sequenced this gene in 349 inbred lines, 68 landraces, and 32 teosintes. Sequence polymorphisms, nucleotide diversity, and neutral tests revealed that *ZmPGP1* might be selected during domestication and improvement processes. Marker–trait association analysis identified 11 variants significantly associated with 4 plant architecture and 5 ear traits, revealing that significant variants in *ZmPGP1* can be used to develop functional markers to improve plant architecture and ear traits in maize [10].

Another particular approach was reported for two popular fruit crops such as strawberry (*Fragaria × ananassa* Duchesne) and raspberry (*Rubus idaeus* L.). Here, Lebedev et al. (2020) [11] reported the potential transferability between the species of a large SSR panel for their employment in breeding programs assisted by functional DNA markers [11]. One hundred eighteen (118) microsatellite loci in the flavonoid biosynthesis were developed to assess the genetic diversity of 48 *Fragaria* and *Rubus* accessions, including wild species and rare cultivars, which differ in berry color, ploidy, and origin. SSR panel may be a useful molecular tool in strawberry and raspberry breeding programs for improvement anthocyanin related traits [11].

Informative molecular markers such as SSR were adopted also for detecting hybridity and homozygosity in breeding segregant populations in lettuce (*Lactuca sativa* L.). In this study, a panel of 16 SSR was used to genotype 71 putative parental lines and to plan 89 controlled crosses designed to maximize the genetic recombination [12]. Unexpected genotypes were detected (5%), consistent with this species’ spontaneous out-pollination rate. Overall, the synergistic advantages of conventional and molecular selection applied in different steps of a breeding programs aimed at developing new varieties were demonstrated [12].

Two other manuscripts reported the usefulness of molecular analysis for analyzing germplasm collections [13,14]. In the first, an SSR panel was adopted to analyze the official Algerian olive (*Olea europea* L.) collection highlighting a biodiversity hotspot in the Mediterranean Basin [13]. The olive germplasm was characterized using 16 nuclear (nuSSR) and six chloroplast (cpSSR) microsatellites, useful to underline the presence of an exclusive genetic core represented by 13 cultivars located in a mountainous area in the North-East of Algeria, named Little Kabylie. The genetic relationship of Algerian and Mediterranean olive germplasm was assessed, suggesting possible events of secondary domestication and/or crossing and hybridization across the Mediterranean area [13]. The second manuscript described the genetic diversity in sweet potato (*Ipomea batatas* L.) genetic resources by morphological and molecular markers [14]. The EU market of this orphan crop has recently increased by 100%, and its cultivation in southern European countries is a new opportunity for the EU to exploit and introduce new genotypes. In this view, the origins of the principal Italian sweet potato clones, compared with a core collection of genotypes from Central and Southern America, were investigated by combining genetic analysis with morphological and chemical measurements [14]. Overall, these markers combination resulted as being effective to cluster the sweet potato clones in agreement with their geographical origin [14]. The last report included in this Special Issue focused on molecular markers supporting the in situ conservation of faba bean (*Vicia faba* L.) landraces in Tunisia [15]. The seed phenotypic features of the collected samples were analyzed, together with the genetic diversity and population structure, by using simple sequence repeat markers, highlighting the genetic stability of the population under study. These findings suggested that farmers applied international best practices for the in situ conservation of agricultural crops [15].

In conclusion, the Special Issue focused on the development and application of such technologies associated with adaptation and functional crop improvement, exploiting the available biodiversity in very different crops, from vegetables and legumes (eggplant, lettuce, sweet potato and faba bean), through important cereals (barley and corn) to very important Mediterranean trees (olive). This issue has allowed a scientific journey through the use of consolidated molecular markers, such as SSR, as well as novel classes of molecular markers obtainable by the new technologies (NGS). These were applied at the genetic analysis of germplasm collections, but also to the findings of new markers and QTL for assisted breeding programs.
